# 1-(4-Methoxy­phenyl­sulfon­yl)-5-methyl-5-phenyl­imidazolidine-2,4-dione

**DOI:** 10.1107/S1600536809016092

**Published:** 2009-05-07

**Authors:** Abid Hussain, Shahid Hameed, Helen Stoeckli-Evans

**Affiliations:** a Department of Chemistry, Quaid-I-Azam University, Islamabad 45320, Pakistan; b Institute of Physics, University of Neuchâtel, rue Emile-Argand 11, CH-2009 Neuchâtel, Switzerland

## Abstract

The title compound, C_17_H_16_N_2_O_5_S, crystallized in the chiral monoclinic space group *P*2_1_, with two enanti­omeric mol­ecules (*A* and *B*) in the asymmetric unit. It is composed of a methyl­imidazolidine-2,4-dione unit substituted with a phenyl group and a 4-methoxy­phenyl­sulfonyl group. The benzene ring mean planes are inclined to one another by 22.20 (14)° in mol­ecule *A* and by 15.82 (13)° in mol­ecule *B*. In the crystal structure, the *A* and *B* mol­ecules are linked by N—H⋯O hydrogen bonds, forming centrosymmetric dimers. A number of C—H⋯O inter­actions are also present in the crystal structure, leading to the formation of a three-dimensinoal network.

## Related literature

For the applications of immidazolidine-2,4-diones, see: Thenmozhiyal *et al.* (2004 ▶); Zhang *et al.* (2004 ▶). For the activity of sulfonyl­immidazolidine-2,4-diones, see: Kashif, Ahmad & Hameed (2008 ▶); Ahmad *et al.* (2000 ▶, 2002 ▶); Murakami *et al.* (1997 ▶). For related crystal structures, see: Hussain *et al.* (2009 ▶); Kashif, Hussain *et al.* (2008 ▶).
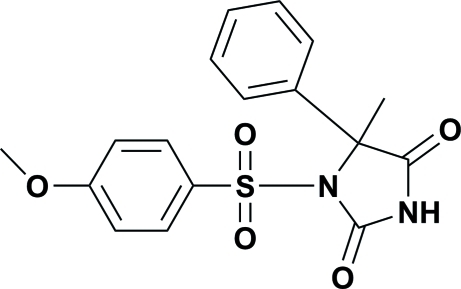


## Experimental

### 

#### Crystal data

C_17_H_16_N_2_O_5_S
*M* *_r_* = 360.38
Monoclinic, 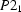

*a* = 6.2314 (6) Å
*b* = 17.5694 (12) Å
*c* = 15.5892 (16) Å
β = 99.373 (12)°
*V* = 1683.9 (3) Å^3^
*Z* = 4
Mo *K*α radiation
μ = 0.22 mm^−1^
*T* = 173 K
0.38 × 0.30 × 0.19 mm

#### Data collection

Stoe IPDS diffractometer
Absorption correction: none
13521 measured reflections
6394 independent reflections
4309 reflections with *I* > 2σ(*I*)
*R*_int_ = 0.033

#### Refinement

*R*[*F* ^2^ > 2σ(*F* ^2^)] = 0.029
*wR*(*F* ^2^) = 0.058
*S* = 0.81
6394 reflections
465 parameters
3 restraints
H atoms treated by a mixture of independent and constrained refinement
Δρ_max_ = 0.17 e Å^−3^
Δρ_min_ = −0.20 e Å^−3^
Absolute structure: Flack (1983 ▶), 2987 Friedel pairs
Flack parameter: 0.06 (5)

### 

Data collection: *EXPOSE* in *IPDS-I* (Stoe & Cie, 2000 ▶); cell refinement: *CELL* in *IPDS-I*; data reduction: *INTEGRATE* in *IPDS-I*; program(s) used to solve structure: *SHELXS97* (Sheldrick, 2008 ▶); program(s) used to refine structure: *SHELXL97* (Sheldrick, 2008 ▶); molecular graphics: *PLATON* (Spek, 2009 ▶); software used to prepare material for publication: *SHELXL97*.

## Supplementary Material

Crystal structure: contains datablocks I, global. DOI: 10.1107/S1600536809016092/bt2941sup1.cif

Structure factors: contains datablocks I. DOI: 10.1107/S1600536809016092/bt2941Isup2.hkl

Additional supplementary materials:  crystallographic information; 3D view; checkCIF report

## Figures and Tables

**Table 1:** Hydrogen-bond geometry (Å, °)

*D*—H⋯*A*	*D*—H	H⋯*A*	*D*⋯*A*	*D*—H⋯*A*
N2—H2*N*⋯O8^i^	0.91 (2)	1.95 (2)	2.851 (3)	171 (2)
N4—H4*N*⋯O3^ii^	0.92 (2)	1.89 (2)	2.800 (3)	171 (2)
C5—H5⋯O2^i^	0.95	2.36	3.218 (3)	150
C10—H10*A*⋯O8	0.98	2.56	3.437 (3)	149
C10—H10*B*⋯O2	0.98	2.45	3.055 (3)	120
C12—H12⋯O2	0.95	2.52	2.905 (3)	104
C13—H13⋯O9^iii^	0.95	2.43	3.287 (3)	150
C22—H22⋯O7^ii^	0.95	2.42	3.351 (3)	165
C24—H24⋯O8^iv^	0.95	2.56	3.492 (3)	166
C27—H27*A*⋯O7	0.98	2.52	3.126 (4)	120
C29—H29⋯O7	0.95	2.54	2.916 (3)	104
C30—H30⋯O4^v^	0.95	2.38	3.159 (3)	138
C34—H34⋯O8	0.95	2.56	3.291 (3)	134

Symmetry codes: (i) 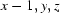; (ii) 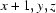; (iii) 
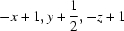
; (iv) 
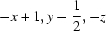
; (v) 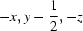.

**Fig. 1.**
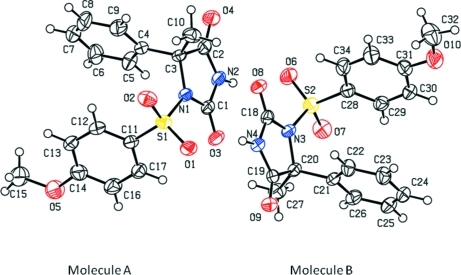
A view of the molecular structure of the two indepenent molecules of the title compound. Displacement ellipsoids are drawn at the 50% probabiltiy level.

**Fig. 2.**
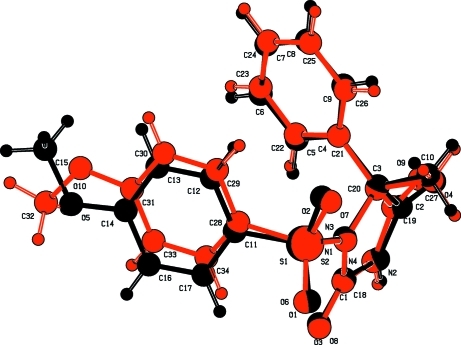
An auto-fit view (Spek, 2009) of the two independent molecules (A black; B red) in the title compound.

**Fig. 3.**
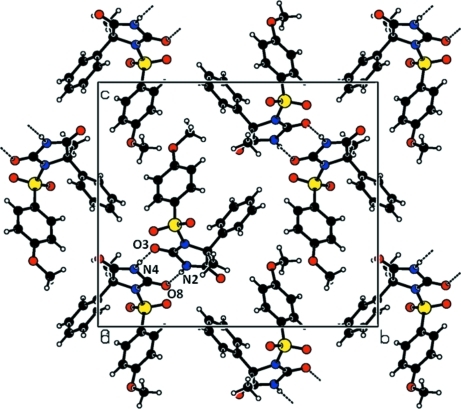
A view along the a axis of the crystal packing in the title compound, showing the N—H···O hydrogen bonds which link the two independent molecules (A and B) to form a dimer-like arrangement (see Table 1 for details).

**Fig. 4.**
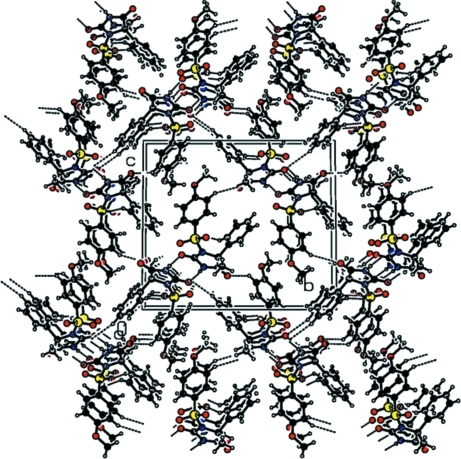
A perspective view along the a axis of the crystal packing in the title compound showing the N—H···O and C—H···O hydrogen bonds, which link the molecules to form a three-dimensional network (see Table 1 for details).

## supplementary crystallographic information

### Comment

Imidazolidine-2,4-diones have found applications as androgen receptor antagonists (Zhang *et al.*, 2004) and possess strong anticonvulsant activity (Thenmozhiyal *et al.*, 2004). Sulfonyl derivatives of imidazolidine-2,4-diones on the other hand, are finding utility as inhibitors of aldose reductase (Murakami *et al.*, 1997). The potential of this class of compounds as hypoglycemic agents has already been reported from our laboratory (Kashif, Ahmad & Hameed, 2008; Ahmad *et al.*, 2002, 2000). In continuation of our work on sulfonyl cyclic ureas (Hussain *et al.*, 2009; Kashif, Hussain *et al.*, 2008), the title compound was synthesized taking an imidazolidine-2,4-dione as the scaffold.

The molecular structure of the title compound is illustrated in Fig. 1, and full geometrical details are available in the archived CIF. It crystallized in the chiral monoclinic space group *P*2_1_ with two enantiomeric molecues (A and B) in the asymmetric unit. It is composed of a methylimidazolidine-2,4-dione moiety substituted with a phenyl group and a 4-methoxyphenylsulfonyl group. The bond distances and angles of the two independent molecules are very similar to those observed in 1-(4-Chlorophenylsulfonyl)-5-(4-fluorophenyl)-5-methylimidazolidine-2,4-dione (Hussain *et al.*, 2009), which also crystallized with two independent molecules per asymmetric unit but in the centrosymmetric triclinic space group P-1.

Both molecules A and B are U-shaped with slightly different conformations, as can be seen in the auto-fit view, Fig. 2 (Spek, 2009). The best fit was obtained for inverted molecule B on molecule A (25 non-H atoms) with the weighted and unit weight r.m.s. fits being 0.687 and 0.460 Å, respectively. The benzene ring mean planes are inclined to one another by 22.20 (14) ° in molecule A and 15.82 (13) ° in molecule B. This is different to the situation in the compound mentioned above where the same angles are 6.07 (8) and 8.67 (8) °, for molecules A and B, respectively (Hussain *et al.*, 2009).

In the crystal structure of the title compound the A and B molecules are linked by N—H···O hydrogen bonds to form a dimer-like arrangement (Fig. 3 and Table 1). There are also a number of C—H···O interactions present in the crystal structure, which leads to the formation of a three-dimensional network (Fig. 4 and Table 1).

### Experimental

5-Methyl-5-phenylimidazolidine-2,4-dione (4.8 mmol) in CH_2_Cl_2_ was stirred with triethyl amine (4.8 mmol) and catalytic amounts of DMAP. 4-Methoxybenzene sulfonyl chloride (5.8 mmol) in CH_2_Cl_2_ was added dropwise. The reaction mixture was stirred continually at 298 K until the reaction was complete (monitored by TLC). The reaction mixture was then diluted with 1 N HCl and extracted with CH_2_Cl_2_ (3 × 25 ml). The organic layer was dried over anhydrous sodium sulfate and concentrated under reduced pressure. Crystallization of the residue in ethyl acetate afforded colourless rod-like crystals of the title compound, suitable for X-ray analysis.

### Refinement

The NH H-atoms were located in difference Fourier maps and freely refined. The H-atoms bonded to C were included in calculated positions [C—H = 0.95 - 0.98 Å] and treated as riding  with *U*_iso_(H) = 1.2*U*_eq_(C) or 1.5*U*_eq_(C_methyl_).

### Crystal data

**Table:**

C_17_H_16_N_2_O_5_S	*F*(000) = 752
*M**_r_* = 360.38	*D*_x_ = 1.421 Mg m^−^^3^
Monoclinic, *P*2_1_	Melting point = 471–473 K
Hall symbol: P 2yb	Mo *K*α radiation, λ = 0.71073 Å
*a* = 6.2314 (6) Å	Cell parameters from 8000 reflections
*b* = 17.5694 (12) Å	θ = 2.6–26.0°
*c* = 15.5892 (16) Å	µ = 0.22 mm^−^^1^
β = 99.373 (12)°	*T* = 173 K
*V* = 1683.9 (3) Å^3^	Rod, colourless
*Z* = 4	0.38 × 0.30 × 0.19 mm

### Data collection

**Table:**

Stoe IPDS diffractometer	4309 reflections with *I* > 2σ(*I*)
Radiation source: fine-focus sealed tube	*R*_int_ = 0.033
graphite	θ_max_ = 26.1°, θ_min_ = 2.7°
φ rotation scans	*h* = −7→7
13521 measured reflections	*k* = −20→21
6394 independent reflections	*l* = −19→19

### Refinement

**Table:**

Refinement on *F*^2^	Hydrogen site location: inferred from neighbouring sites
Least-squares matrix: full	H atoms treated by a mixture of independent and constrained refinement
*R*[*F*^2^ > 2σ(*F*^2^)] = 0.029	*w* = 1/[σ^2^(*F*_o_^2^) + (0.024*P*)^2^] where *P* = (*F*_o_^2^ + 2*F*_c_^2^)/3
*wR*(*F*^2^) = 0.058	(Δ/σ)_max_ = 0.001
*S* = 0.81	Δρ_max_ = 0.17 e Å^−^^3^
6394 reflections	Δρ_min_ = −0.20 e Å^−^^3^
465 parameters	Extinction correction: *SHELXL97* (Sheldrick, 2008), Fc^*^=kFc[1+0.001xFc^2^λ^3^/sin(2θ)]^-1/4^
3 restraints	Extinction coefficient: 0.0042 (4)
Primary atom site location: structure-invariant direct methods	Absolute structure: Flack (1983), 2987 Friedel pairs
Secondary atom site location: difference Fourier map	Flack parameter: 0.06 (5)

### Special details

**Table:**

Geometry. Bond distances, angles *etc*. have been calculated using the rounded fractional coordinates. All su's are estimated from the variances of the (full) variance-covariance matrix. The cell e.s.d.'s are taken into account in the estimation of distances, angles and torsion angles
Refinement. Refinement of *F*^2^ against ALL reflections. The weighted *R*-factor *wR* and goodness of fit *S* are based on *F*^2^, conventional *R*-factors *R* are based on *F*, with *F* set to zero for negative *F*^2^. The threshold expression of *F*^2^ > σ(*F*^2^) is used only for calculating *R*-factors(gt) *etc*. and is not relevant to the choice of reflections for refinement. *R*-factors based on *F*^2^ are statistically about twice as large as those based on *F*, and *R*- factors based on ALL data will be even larger.

### Fractional atomic coordinates and isotropic or equivalent isotropic displacement parameters (Å^2^)

**Table:**

	*x*	*y*	*z*	*U*_iso_*/*U*_eq_	
S1	0.49288 (9)	0.27479 (4)	0.41621 (4)	0.0322 (2)	
O1	0.5317 (3)	0.19861 (10)	0.39102 (11)	0.0374 (6)	
O2	0.6619 (3)	0.33014 (11)	0.42511 (11)	0.0414 (6)	
O3	0.0791 (3)	0.20334 (11)	0.32063 (11)	0.0388 (6)	
O4	−0.0034 (3)	0.43832 (11)	0.19756 (12)	0.0497 (7)	
O5	0.0979 (3)	0.26824 (12)	0.73090 (12)	0.0622 (8)	
N1	0.3022 (3)	0.30995 (11)	0.33861 (12)	0.0273 (7)	
N2	−0.0122 (3)	0.31655 (12)	0.24927 (13)	0.0340 (8)	
C1	0.1190 (4)	0.26883 (16)	0.30444 (15)	0.0299 (8)	
C2	0.0728 (4)	0.38756 (16)	0.24475 (17)	0.0330 (9)	
C3	0.2862 (4)	0.39125 (14)	0.30845 (15)	0.0285 (8)	
C4	0.2584 (4)	0.44517 (15)	0.38246 (15)	0.0289 (8)	
C5	0.0795 (4)	0.43753 (17)	0.42345 (17)	0.0409 (10)	
C6	0.0477 (5)	0.48556 (18)	0.49017 (19)	0.0480 (10)	
C7	0.1927 (5)	0.54235 (18)	0.51559 (19)	0.0519 (11)	
C8	0.3705 (6)	0.55014 (19)	0.4763 (2)	0.0616 (11)	
C9	0.4043 (5)	0.50230 (18)	0.40945 (18)	0.0483 (10)	
C10	0.4663 (4)	0.41178 (16)	0.25751 (18)	0.0419 (10)	
C11	0.3765 (4)	0.27369 (15)	0.51029 (15)	0.0303 (8)	
C12	0.4167 (4)	0.33274 (15)	0.56842 (16)	0.0395 (9)	
C13	0.3248 (5)	0.33334 (16)	0.64261 (17)	0.0447 (10)	
C14	0.1936 (4)	0.27364 (18)	0.65885 (16)	0.0438 (10)	
C15	0.1345 (7)	0.32823 (19)	0.7938 (2)	0.0826 (16)	
C16	0.1511 (4)	0.21413 (17)	0.60013 (18)	0.0453 (10)	
C17	0.2418 (4)	0.21460 (15)	0.52550 (16)	0.0360 (9)	
S2	0.17941 (9)	0.16666 (4)	0.07688 (4)	0.0343 (2)	
O6	0.1557 (3)	0.24522 (10)	0.09316 (11)	0.0443 (7)	
O7	0.0040 (3)	0.11541 (11)	0.07948 (12)	0.0434 (6)	
O8	0.5996 (3)	0.24274 (10)	0.16846 (11)	0.0382 (6)	
O9	0.7248 (3)	0.00425 (10)	0.27716 (11)	0.0390 (6)	
O10	0.5175 (3)	0.11964 (11)	−0.24698 (12)	0.0523 (7)	
N3	0.3788 (3)	0.13476 (12)	0.15370 (13)	0.0285 (7)	
N4	0.7059 (3)	0.12965 (12)	0.23550 (12)	0.0295 (7)	
C18	0.5652 (4)	0.17648 (16)	0.18420 (15)	0.0290 (8)	
C19	0.6295 (4)	0.05719 (16)	0.23806 (15)	0.0300 (8)	
C20	0.4033 (4)	0.05427 (15)	0.18445 (15)	0.0286 (8)	
C21	0.4028 (4)	−0.00248 (14)	0.10977 (15)	0.0284 (8)	
C22	0.5752 (4)	−0.00181 (16)	0.06382 (15)	0.0354 (9)	
C23	0.5797 (4)	−0.05236 (18)	−0.00387 (17)	0.0442 (10)	
C24	0.4127 (5)	−0.10347 (16)	−0.02589 (17)	0.0466 (10)	
C25	0.2398 (5)	−0.10367 (18)	0.01867 (19)	0.0488 (11)	
C26	0.2350 (4)	−0.05315 (16)	0.08642 (17)	0.0390 (9)	
C27	0.2452 (5)	0.03531 (17)	0.24687 (19)	0.0424 (10)	
C28	0.2780 (4)	0.15486 (15)	−0.02036 (16)	0.0318 (8)	
C29	0.1934 (4)	0.09875 (15)	−0.07862 (16)	0.0338 (8)	
C30	0.2755 (4)	0.08912 (15)	−0.15410 (16)	0.0366 (9)	
C31	0.4463 (4)	0.13473 (15)	−0.17102 (16)	0.0375 (9)	
C32	0.6969 (5)	0.1640 (2)	−0.26728 (19)	0.0633 (13)	
C33	0.5329 (4)	0.18981 (17)	−0.11248 (17)	0.0487 (10)	
C34	0.4462 (4)	0.19955 (17)	−0.03783 (17)	0.0453 (10)	
H2N	−0.143 (3)	0.2979 (16)	0.2234 (17)	0.057 (9)*	
H5	−0.02360	0.39850	0.40540	0.0490*	
H6	−0.07520	0.47900	0.51840	0.0580*	
H7	0.16960	0.57630	0.56060	0.0620*	
H8	0.47330	0.58910	0.49500	0.0740*	
H9	0.52860	0.50890	0.38210	0.0580*	
H10A	0.47220	0.37370	0.21200	0.0630*	
H10B	0.60600	0.41290	0.29690	0.0630*	
H10C	0.43690	0.46200	0.23070	0.0630*	
H12	0.50880	0.37340	0.55720	0.0470*	
H13	0.35120	0.37450	0.68240	0.0540*	
H15A	0.29050	0.33220	0.81610	0.1240*	
H15B	0.08230	0.37640	0.76630	0.1240*	
H15C	0.05580	0.31710	0.84180	0.1240*	
H16	0.05990	0.17330	0.61140	0.0540*	
H17	0.21190	0.17440	0.48450	0.0430*	
H4N	0.835 (3)	0.1490 (15)	0.2635 (16)	0.053 (9)*	
H22	0.69070	0.03350	0.07890	0.0420*	
H23	0.69830	−0.05180	−0.03520	0.0530*	
H24	0.41700	−0.13860	−0.07190	0.0560*	
H25	0.12350	−0.13850	0.00300	0.0590*	
H26	0.11500	−0.05340	0.11700	0.0470*	
H27A	0.09600	0.03590	0.21510	0.0640*	
H27B	0.25970	0.07320	0.29360	0.0640*	
H27C	0.27880	−0.01530	0.27190	0.0640*	
H29	0.07880	0.06710	−0.06620	0.0410*	
H30	0.21600	0.05140	−0.19480	0.0440*	
H32A	0.82400	0.15630	−0.22210	0.0950*	
H32B	0.65720	0.21800	−0.27000	0.0950*	
H32C	0.73170	0.14780	−0.32360	0.0950*	
H33	0.65070	0.22050	−0.12370	0.0580*	
H34	0.50350	0.23790	0.00250	0.0540*	

### Atomic displacement parameters (Å^2^)

**Table:**

	*U*^11^	*U*^22^	*U*^33^	*U*^12^	*U*^13^	*U*^23^
S1	0.0318 (3)	0.0308 (4)	0.0350 (3)	0.0037 (3)	0.0081 (3)	0.0046 (3)
O1	0.0456 (10)	0.0279 (11)	0.0406 (10)	0.0154 (8)	0.0127 (8)	0.0030 (8)
O2	0.0309 (9)	0.0458 (13)	0.0475 (11)	−0.0090 (9)	0.0062 (8)	0.0103 (10)
O3	0.0469 (10)	0.0229 (12)	0.0444 (11)	−0.0020 (8)	0.0006 (8)	−0.0035 (9)
O4	0.0650 (12)	0.0332 (12)	0.0448 (11)	0.0118 (10)	−0.0093 (9)	0.0037 (10)
O5	0.0954 (15)	0.0495 (14)	0.0531 (12)	0.0007 (12)	0.0464 (11)	−0.0022 (11)
N1	0.0297 (10)	0.0221 (13)	0.0300 (11)	0.0007 (8)	0.0046 (9)	−0.0009 (9)
N2	0.0392 (13)	0.0240 (14)	0.0355 (12)	0.0028 (10)	−0.0037 (10)	−0.0047 (10)
C1	0.0356 (13)	0.0231 (16)	0.0305 (13)	0.0035 (13)	0.0038 (10)	−0.0040 (12)
C2	0.0422 (15)	0.0260 (17)	0.0308 (15)	0.0074 (12)	0.0060 (12)	−0.0041 (12)
C3	0.0352 (13)	0.0192 (15)	0.0315 (14)	0.0022 (10)	0.0068 (11)	0.0020 (11)
C4	0.0350 (13)	0.0224 (15)	0.0289 (13)	0.0001 (11)	0.0043 (11)	0.0009 (11)
C5	0.0381 (15)	0.0392 (19)	0.0462 (17)	−0.0060 (13)	0.0092 (13)	−0.0137 (14)
C6	0.0500 (17)	0.055 (2)	0.0410 (16)	0.0091 (15)	0.0136 (13)	−0.0104 (14)
C7	0.083 (2)	0.035 (2)	0.0374 (16)	0.0077 (16)	0.0091 (16)	−0.0062 (14)
C8	0.091 (2)	0.046 (2)	0.0474 (19)	−0.0311 (18)	0.0097 (18)	−0.0166 (16)
C9	0.0581 (17)	0.041 (2)	0.0479 (17)	−0.0172 (15)	0.0152 (14)	−0.0016 (15)
C10	0.0473 (16)	0.0388 (19)	0.0435 (16)	0.0055 (13)	0.0187 (13)	0.0089 (13)
C11	0.0329 (12)	0.0241 (15)	0.0346 (13)	−0.0004 (12)	0.0078 (10)	0.0022 (12)
C12	0.0529 (16)	0.0253 (17)	0.0408 (15)	−0.0063 (12)	0.0091 (13)	0.0031 (13)
C13	0.0721 (19)	0.0257 (17)	0.0390 (15)	−0.0021 (14)	0.0175 (14)	−0.0040 (12)
C14	0.0571 (17)	0.0390 (18)	0.0404 (15)	0.0067 (15)	0.0234 (13)	0.0032 (14)
C15	0.154 (4)	0.048 (2)	0.060 (2)	0.012 (2)	0.060 (2)	−0.0070 (18)
C16	0.0553 (17)	0.0359 (19)	0.0496 (17)	−0.0069 (13)	0.0235 (13)	−0.0008 (14)
C17	0.0407 (14)	0.0306 (17)	0.0384 (14)	−0.0018 (12)	0.0117 (11)	−0.0037 (12)
S2	0.0317 (3)	0.0336 (4)	0.0382 (4)	0.0030 (3)	0.0073 (3)	−0.0010 (3)
O6	0.0518 (11)	0.0318 (13)	0.0487 (11)	0.0145 (9)	0.0062 (9)	−0.0040 (9)
O7	0.0269 (9)	0.0545 (13)	0.0501 (11)	−0.0038 (9)	0.0101 (8)	0.0012 (10)
O8	0.0482 (10)	0.0232 (12)	0.0417 (11)	−0.0054 (8)	0.0026 (8)	−0.0007 (8)
O9	0.0500 (10)	0.0287 (12)	0.0367 (10)	0.0005 (9)	0.0024 (8)	0.0032 (9)
O10	0.0612 (12)	0.0570 (15)	0.0427 (11)	−0.0077 (10)	0.0202 (9)	−0.0060 (10)
N3	0.0325 (10)	0.0199 (13)	0.0335 (11)	−0.0013 (8)	0.0064 (9)	−0.0012 (9)
N4	0.0337 (12)	0.0226 (13)	0.0320 (12)	−0.0019 (10)	0.0050 (9)	−0.0015 (9)
C18	0.0330 (13)	0.0269 (17)	0.0284 (13)	0.0023 (12)	0.0085 (10)	−0.0048 (12)
C19	0.0430 (15)	0.0258 (16)	0.0231 (13)	−0.0004 (12)	0.0109 (11)	−0.0014 (11)
C20	0.0325 (13)	0.0228 (15)	0.0325 (14)	−0.0023 (10)	0.0114 (11)	−0.0041 (11)
C21	0.0323 (13)	0.0204 (15)	0.0324 (14)	−0.0014 (11)	0.0050 (11)	−0.0017 (11)
C22	0.0345 (13)	0.0394 (18)	0.0334 (14)	−0.0009 (12)	0.0090 (11)	−0.0068 (12)
C23	0.0547 (17)	0.0437 (19)	0.0353 (15)	0.0077 (15)	0.0105 (13)	−0.0057 (14)
C24	0.077 (2)	0.0272 (18)	0.0341 (15)	0.0070 (15)	0.0050 (15)	−0.0063 (13)
C25	0.0674 (19)	0.0307 (19)	0.0450 (17)	−0.0152 (14)	−0.0007 (15)	−0.0053 (14)
C26	0.0448 (15)	0.0308 (17)	0.0421 (16)	−0.0109 (13)	0.0088 (12)	−0.0017 (13)
C27	0.0479 (16)	0.0352 (19)	0.0494 (18)	−0.0030 (13)	0.0242 (14)	−0.0004 (13)
C28	0.0326 (12)	0.0291 (16)	0.0331 (13)	0.0000 (11)	0.0037 (10)	0.0007 (11)
C29	0.0328 (13)	0.0308 (16)	0.0371 (14)	−0.0056 (11)	0.0033 (11)	0.0006 (12)
C30	0.0426 (15)	0.0297 (17)	0.0360 (15)	−0.0056 (12)	0.0023 (12)	−0.0042 (12)
C31	0.0429 (14)	0.0403 (18)	0.0303 (14)	−0.0028 (13)	0.0088 (11)	0.0005 (12)
C32	0.0559 (18)	0.088 (3)	0.0501 (17)	−0.0130 (19)	0.0212 (14)	0.0026 (18)
C33	0.0520 (16)	0.055 (2)	0.0409 (16)	−0.0264 (14)	0.0131 (13)	−0.0059 (14)
C34	0.0553 (17)	0.0441 (19)	0.0362 (15)	−0.0192 (14)	0.0069 (13)	−0.0085 (13)

### Geometric parameters (Å, °)

**Table:**

S1—O1	1.4266 (19)	C7—H7	0.9500
S1—O2	1.424 (2)	C8—H8	0.9500
S1—N1	1.670 (2)	C9—H9	0.9500
S1—C11	1.739 (2)	C10—H10B	0.9800
S2—N3	1.676 (2)	C10—H10A	0.9800
S2—O6	1.4155 (19)	C10—H10C	0.9800
S2—O7	1.422 (2)	C12—H12	0.9500
S2—C28	1.738 (3)	C13—H13	0.9500
O3—C1	1.212 (3)	C15—H15B	0.9800
O4—C2	1.203 (3)	C15—H15A	0.9800
O5—C14	1.358 (3)	C15—H15C	0.9800
O5—C15	1.432 (4)	C16—H16	0.9500
O8—C18	1.216 (3)	C17—H17	0.9500
O9—C19	1.213 (3)	C19—C20	1.518 (3)
O10—C31	1.357 (3)	C20—C27	1.530 (4)
O10—C32	1.440 (4)	C20—C21	1.532 (3)
N1—C1	1.382 (3)	C21—C22	1.385 (3)
N1—C3	1.502 (3)	C21—C26	1.376 (4)
N2—C1	1.372 (3)	C22—C23	1.383 (4)
N2—C2	1.362 (3)	C23—C24	1.375 (4)
N2—H2N	0.91 (2)	C24—C25	1.374 (4)
N3—C20	1.493 (3)	C25—C26	1.384 (4)
N3—C18	1.390 (3)	C28—C34	1.372 (4)
N4—C18	1.363 (3)	C28—C29	1.385 (4)
N4—C19	1.362 (3)	C29—C30	1.368 (4)
N4—H4N	0.92 (2)	C30—C31	1.391 (4)
C2—C3	1.526 (4)	C31—C33	1.378 (4)
C3—C4	1.524 (3)	C33—C34	1.371 (4)
C3—C10	1.520 (4)	C22—H22	0.9500
C4—C9	1.374 (4)	C23—H23	0.9500
C4—C5	1.379 (4)	C24—H24	0.9500
C5—C6	1.379 (4)	C25—H25	0.9500
C6—C7	1.361 (4)	C26—H26	0.9500
C7—C8	1.357 (5)	C27—H27A	0.9800
C8—C9	1.381 (4)	C27—H27B	0.9800
C11—C17	1.380 (4)	C27—H27C	0.9800
C11—C12	1.373 (4)	C29—H29	0.9500
C12—C13	1.372 (4)	C30—H30	0.9500
C13—C14	1.378 (4)	C32—H32A	0.9800
C14—C16	1.387 (4)	C32—H32B	0.9800
C16—C17	1.374 (4)	C32—H32C	0.9800
C5—H5	0.9500	C33—H33	0.9500
C6—H6	0.9500	C34—H34	0.9500
			
O1—S1—O2	120.78 (12)	C13—C12—H12	120.00
O1—S1—N1	106.27 (10)	C14—C13—H13	120.00
O1—S1—C11	109.54 (12)	C12—C13—H13	120.00
O2—S1—N1	104.23 (11)	O5—C15—H15B	109.00
O2—S1—C11	109.21 (12)	O5—C15—H15A	109.00
N1—S1—C11	105.65 (11)	H15A—C15—H15B	109.00
O7—S2—N3	104.59 (11)	H15A—C15—H15C	110.00
O7—S2—C28	109.38 (12)	O5—C15—H15C	109.00
N3—S2—C28	104.90 (11)	H15B—C15—H15C	110.00
O6—S2—C28	109.55 (12)	C14—C16—H16	120.00
O6—S2—O7	120.61 (12)	C17—C16—H16	120.00
O6—S2—N3	106.56 (11)	C16—C17—H17	120.00
C14—O5—C15	118.2 (2)	C11—C17—H17	120.00
C31—O10—C32	117.5 (2)	N3—C18—N4	107.7 (2)
S1—N1—C1	122.10 (17)	O8—C18—N3	126.9 (2)
S1—N1—C3	125.57 (16)	O8—C18—N4	125.4 (2)
C1—N1—C3	111.58 (19)	O9—C19—C20	125.8 (2)
C1—N2—C2	113.3 (2)	O9—C19—N4	125.9 (2)
C2—N2—H2N	129.4 (18)	N4—C19—C20	108.3 (2)
C1—N2—H2N	117.2 (18)	N3—C20—C21	112.64 (19)
S2—N3—C18	123.31 (18)	N3—C20—C19	100.5 (2)
S2—N3—C20	124.85 (16)	C19—C20—C27	107.1 (2)
C18—N3—C20	110.71 (19)	C21—C20—C27	114.8 (2)
C18—N4—C19	112.8 (2)	N3—C20—C27	111.6 (2)
C18—N4—H4N	119.1 (16)	C19—C20—C21	109.1 (2)
C19—N4—H4N	128.1 (16)	C22—C21—C26	119.3 (2)
N1—C1—N2	107.0 (2)	C20—C21—C22	118.8 (2)
O3—C1—N1	126.9 (2)	C20—C21—C26	122.0 (2)
O3—C1—N2	126.1 (2)	C21—C22—C23	120.2 (2)
O4—C2—C3	125.6 (2)	C22—C23—C24	120.1 (2)
O4—C2—N2	126.3 (2)	C23—C24—C25	120.0 (3)
N2—C2—C3	108.1 (2)	C24—C25—C26	120.0 (3)
N1—C3—C10	111.9 (2)	C21—C26—C25	120.5 (2)
N1—C3—C4	111.35 (19)	C29—C28—C34	119.9 (2)
N1—C3—C2	99.77 (19)	S2—C28—C29	120.4 (2)
C2—C3—C4	108.9 (2)	S2—C28—C34	119.7 (2)
C2—C3—C10	108.0 (2)	C28—C29—C30	119.8 (2)
C4—C3—C10	115.6 (2)	C29—C30—C31	119.8 (2)
C5—C4—C9	118.3 (2)	O10—C31—C33	124.5 (2)
C3—C4—C9	122.3 (2)	O10—C31—C30	115.0 (2)
C3—C4—C5	119.4 (2)	C30—C31—C33	120.5 (2)
C4—C5—C6	121.1 (3)	C31—C33—C34	118.9 (2)
C5—C6—C7	119.9 (3)	C28—C34—C33	121.1 (3)
C6—C7—C8	119.6 (3)	C21—C22—H22	120.00
C7—C8—C9	121.0 (3)	C23—C22—H22	120.00
C4—C9—C8	120.1 (3)	C22—C23—H23	120.00
S1—C11—C17	120.15 (19)	C24—C23—H23	120.00
S1—C11—C12	119.5 (2)	C23—C24—H24	120.00
C12—C11—C17	120.4 (2)	C25—C24—H24	120.00
C11—C12—C13	120.4 (2)	C24—C25—H25	120.00
C12—C13—C14	119.4 (3)	C26—C25—H25	120.00
O5—C14—C16	115.5 (3)	C21—C26—H26	120.00
C13—C14—C16	120.5 (2)	C25—C26—H26	120.00
O5—C14—C13	124.0 (3)	C20—C27—H27A	109.00
C14—C16—C17	119.6 (3)	C20—C27—H27B	110.00
C11—C17—C16	119.7 (2)	C20—C27—H27C	109.00
C4—C5—H5	119.00	H27A—C27—H27B	109.00
C6—C5—H5	119.00	H27A—C27—H27C	110.00
C5—C6—H6	120.00	H27B—C27—H27C	109.00
C7—C6—H6	120.00	C28—C29—H29	120.00
C6—C7—H7	120.00	C30—C29—H29	120.00
C8—C7—H7	120.00	C29—C30—H30	120.00
C7—C8—H8	120.00	C31—C30—H30	120.00
C9—C8—H8	119.00	O10—C32—H32A	109.00
C4—C9—H9	120.00	O10—C32—H32B	110.00
C8—C9—H9	120.00	O10—C32—H32C	109.00
H10A—C10—H10B	109.00	H32A—C32—H32B	109.00
H10A—C10—H10C	109.00	H32A—C32—H32C	109.00
C3—C10—H10C	109.00	H32B—C32—H32C	109.00
C3—C10—H10B	109.00	C31—C33—H33	121.00
C3—C10—H10A	110.00	C34—C33—H33	121.00
H10B—C10—H10C	110.00	C28—C34—H34	119.00
C11—C12—H12	120.00	C33—C34—H34	119.00
			
O1—S1—N1—C1	44.6 (2)	N2—C2—C3—N1	4.8 (2)
O1—S1—N1—C3	−146.18 (18)	O4—C2—C3—N1	−173.7 (2)
O2—S1—N1—C1	173.23 (18)	O4—C2—C3—C4	69.6 (3)
O2—S1—N1—C3	−17.6 (2)	O4—C2—C3—C10	−56.8 (3)
C11—S1—N1—C1	−71.7 (2)	C10—C3—C4—C9	−8.8 (4)
C11—S1—N1—C3	97.5 (2)	N1—C3—C4—C9	120.3 (3)
O1—S1—C11—C12	149.2 (2)	C2—C3—C4—C5	48.5 (3)
O1—S1—C11—C17	−31.9 (2)	N1—C3—C4—C5	−60.6 (3)
O2—S1—C11—C12	14.9 (2)	C10—C3—C4—C5	170.3 (2)
O2—S1—C11—C17	−166.3 (2)	C2—C3—C4—C9	−130.6 (3)
N1—S1—C11—C12	−96.7 (2)	C9—C4—C5—C6	−0.4 (4)
N1—S1—C11—C17	82.2 (2)	C3—C4—C5—C6	−179.5 (2)
O6—S2—N3—C18	−38.6 (2)	C3—C4—C9—C8	179.4 (3)
O6—S2—N3—C20	154.74 (18)	C5—C4—C9—C8	0.3 (4)
O7—S2—N3—C18	−167.37 (19)	C4—C5—C6—C7	1.0 (4)
O7—S2—N3—C20	26.0 (2)	C5—C6—C7—C8	−1.5 (5)
C28—S2—N3—C18	77.5 (2)	C6—C7—C8—C9	1.5 (5)
C28—S2—N3—C20	−89.1 (2)	C7—C8—C9—C4	−0.8 (5)
O6—S2—C28—C29	−137.8 (2)	S1—C11—C12—C13	179.4 (2)
O6—S2—C28—C34	44.6 (3)	C17—C11—C12—C13	0.5 (4)
O7—S2—C28—C29	−3.6 (3)	S1—C11—C17—C16	179.8 (2)
O7—S2—C28—C34	178.9 (2)	C12—C11—C17—C16	−1.4 (4)
N3—S2—C28—C29	108.1 (2)	C11—C12—C13—C14	0.8 (4)
N3—S2—C28—C34	−69.4 (2)	C12—C13—C14—O5	178.9 (3)
C15—O5—C14—C13	0.2 (4)	C12—C13—C14—C16	−1.2 (4)
C15—O5—C14—C16	−179.6 (3)	O5—C14—C16—C17	−179.7 (2)
C32—O10—C31—C30	−178.8 (2)	C13—C14—C16—C17	0.4 (4)
C32—O10—C31—C33	0.5 (4)	C14—C16—C17—C11	0.9 (4)
S1—N1—C3—C2	−175.22 (16)	O9—C19—C20—N3	−179.8 (2)
S1—N1—C1—O3	−5.0 (4)	O9—C19—C20—C21	−61.3 (3)
S1—N1—C1—N2	174.13 (15)	O9—C19—C20—C27	63.6 (3)
C3—N1—C1—O3	−175.6 (2)	N4—C19—C20—N3	0.9 (2)
C3—N1—C1—N2	3.6 (3)	N4—C19—C20—C21	119.4 (2)
C1—N1—C3—C2	−5.0 (2)	N4—C19—C20—C27	−115.8 (2)
S1—N1—C3—C4	−60.3 (3)	N3—C20—C21—C22	67.3 (3)
S1—N1—C3—C10	70.7 (2)	N3—C20—C21—C26	−111.8 (3)
C1—N1—C3—C4	109.9 (2)	C19—C20—C21—C22	−43.4 (3)
C1—N1—C3—C10	−119.1 (2)	C19—C20—C21—C26	137.6 (2)
C1—N2—C2—O4	175.3 (3)	C27—C20—C21—C22	−163.5 (2)
C2—N2—C1—O3	179.0 (2)	C27—C20—C21—C26	17.4 (3)
C2—N2—C1—N1	−0.2 (3)	C20—C21—C22—C23	179.9 (2)
C1—N2—C2—C3	−3.2 (3)	C26—C21—C22—C23	−1.0 (4)
S2—N3—C18—O8	9.1 (4)	C20—C21—C26—C25	−180.0 (3)
S2—N3—C18—N4	−170.73 (16)	C22—C21—C26—C25	1.0 (4)
C20—N3—C18—N4	−2.4 (3)	C21—C22—C23—C24	0.1 (4)
S2—N3—C20—C19	169.04 (16)	C22—C23—C24—C25	0.8 (4)
S2—N3—C20—C21	53.1 (3)	C23—C24—C25—C26	−0.9 (4)
S2—N3—C20—C27	−77.7 (2)	C24—C25—C26—C21	−0.1 (4)
C18—N3—C20—C19	0.9 (2)	S2—C28—C29—C30	−178.7 (2)
C18—N3—C20—C21	−115.0 (2)	C34—C28—C29—C30	−1.2 (4)
C18—N3—C20—C27	114.2 (2)	S2—C28—C34—C33	177.6 (2)
C20—N3—C18—O8	177.4 (2)	C29—C28—C34—C33	0.1 (4)
C19—N4—C18—N3	3.1 (3)	C28—C29—C30—C31	1.3 (4)
C18—N4—C19—O9	178.2 (2)	C29—C30—C31—O10	179.0 (2)
C18—N4—C19—C20	−2.5 (3)	C29—C30—C31—C33	−0.3 (4)
C19—N4—C18—O8	−176.8 (2)	O10—C31—C33—C34	180.0 (2)
N2—C2—C3—C4	−112.0 (2)	C30—C31—C33—C34	−0.8 (4)
N2—C2—C3—C10	121.7 (2)	C31—C33—C34—C28	0.9 (4)

### Hydrogen-bond geometry (Å, °)

**Table:**

*D*—H···*A*	*D*—H	H···*A*	*D*···*A*	*D*—H···*A*
N2—H2N···O8^i^	0.91 (2)	1.95 (2)	2.851 (3)	171 (2)
N4—H4N···O3^ii^	0.92 (2)	1.89 (2)	2.800 (3)	171 (2)
C5—H5···O2^i^	0.95	2.36	3.218 (3)	150
C10—H10A···O8	0.98	2.56	3.437 (3)	149
C10—H10B···O2	0.98	2.45	3.055 (3)	120
C12—H12···O2	0.95	2.52	2.905 (3)	104
C13—H13···O9^iii^	0.95	2.43	3.287 (3)	150
C22—H22···O7^ii^	0.95	2.42	3.351 (3)	165
C24—H24···O8^iv^	0.95	2.56	3.492 (3)	166
C27—H27A···O7	0.98	2.52	3.126 (4)	120
C29—H29···O7	0.95	2.54	2.916 (3)	104
C30—H30···O4^v^	0.95	2.38	3.159 (3)	138
C34—H34···O8	0.95	2.56	3.291 (3)	134

Symmetry codes: (i) *x*−1, *y*, *z*; (ii) *x*+1, *y*, *z*; (iii) −*x*+1, *y*+1/2, −*z*+1; (iv) −*x*+1, *y*−1/2, −*z*; (v) −*x*, *y*−1/2, −*z*.
